# Psychosocial difficulties, deprivation and cancer: three questionnaire studies involving 609 cancer patients

**DOI:** 10.1038/sj.bjc.6602777

**Published:** 2005-09-06

**Authors:** P Wright, A Smith, L Booth, A Winterbottom, M Kiely, G Velikova, P Selby

**Affiliations:** 1 Psychosocial and Clinical Practice Research Group, Cancer Research UK Clinical Centre in Leeds, St James's University Hospital, Beckett Street, Leeds LS9 7TF, UK

**Keywords:** social difficulties, psychological distress, deprivation

## Abstract

The aim of the study is to investigate associations between deprivation and self-reported social difficulties and psychological distress in cancer patients. A total of 304 men and 305 women (age range 18–88 years) with a range of cancer diagnoses and living in a socially diverse region (Carstairs and Morris index) completed the Hospital Anxiety and Depression Scale and the Social Difficulties Inventory. Univariate analyses of variance revealed statistically significant differences in reported social difficulties between groups (F (67, 576)=2.4, *P*<0.0001) with stage of disease (F (5, 576)=7.6, *P*<0.0001), age (F (2, 576)=4.8, *P*=0.009) and to a lesser extent deprivation (F (1, 576)=4.0, *P*=0.048) making significant contributions. Significantly more social difficulties were reported by less affluent patients with locally recurrent disease or ‘survivors’. No other interactions were found. Significant differences in levels of reported psychological distress were found between groups (F (67, 575)=1.723, *P*=0.001) for stage of disease, sex and deprivation but no interactions observed. In conclusion, deprivation is associated with reported psychological distress and, to a lesser extent, social difficulties. Patients at particular risk cannot be identified with confidence by socio-demographic and clinical means supporting the recommendation from National Institute for Clinical Excellence for provision of psychosocial assessment for individual cancer patients.

In 2004, the National Institute for Clinical Excellence (NICE) identified social support as one of the main topic areas for improving supportive and palliative care for adults with cancer. Their definition of social support was wide and included: emotional support, help with personal care, advice on employment and financial issues, help in the home, practical aids and adaptations and help to care for dependents (National Institute for Clinical Excellence, 2004). Assessment of social care needs is a key component of the NICE recommendations:

6.18 Teams should ensure that social care needs of each patient are identified as part of initial routine assessment and, are then assessed on an ongoing basis (National Institute for Clinical Excellence, 2004).

Social deprivation is clearly a very important factor in outcomes from cancer. There are consistent associations between deprivation indices and survival for cancer patients across the United Kingdom and although cancer survival overall is improving there is a widening survival gap between the rich and the poor (Coleman *et al*, 2004). The reasons for the association between social deprivation and poor outcomes are complex and little understood but may involve poorer access to transport, being employed in manual work and how people communicate (Dixon *et al*, 2003) and possibly concomitant morbidity (Wrigley *et al*, 2003). These require further study. Deprivation appears to have minimal or no impact on availability of health care (Macleod *et al*, 2000; Campbell *et al*, 2002). Therefore, poorer outcomes for socially deprived people cannot be attributed to inequality in access to health care.

However, it is equally important to ask, in view of the guidance published by NICE, whether social deprivation is a significant indication of perceived psychological and social difficulties among cancer patients. This question is much more difficult to address using Cancer Registries and national databases which, in general, do not collect subjective information from patients on their psychosocial difficulties.

Social difficulties (MORI, 1992; Wright *et al*, 2002) and psychological distress (Stark *et al*, 2002) are common in cancer patients but it is less clear whether these are exclusively the result of the diagnosis of cancer. A widely held view is that most reported perceived difficulties and distress in these domains reflect longstanding issues and circumstances that may not be directly related to the cancer diagnosis and its treatment. Two studies investigating the association between psychological distress and deprivation, one in primary care (Stirling *et al*, 2001) and the second in breast cancer care (Macleod *et al*, 2004), both confirmed higher levels of reported psychological distress in those from poorer communities. This might be expected also for social difficulties but there is a paucity of evidence to inform this view.

The aim of this work is to test the hypothesis that social difficulties and psychological distress are associated with deprivation in a large population of cancer patients in an economically diverse part of the country.

## MATERIALS AND METHODS

From February 2001 to April 2004 we conducted three studies, approved by local research ethics committees:

*Study 1*. A psychometric evaluation of the Social Difficulties Inventory (SDI) in which 270 patients took part in a randomized study involving completion of the SDI using a computer touchscreen followed by randomization into a test–retest arm or home interview arm. Details of this study are published elsewhere (Wright *et al*, 2005).

*Studies 2 (a cross-sectional study) and 3 (a longitudinal study).* These two studies are part of an ongoing project to assess the clinical meaning and utility of the SDI for use in routine oncology practice. In both studies, data were collected from patients attending the Leeds Cancer Centre, a tertiary cancer centre with an approximate 3 million catchment population. Adult patients were recruited from outpatient clinics and wards from haematology, medical oncology, clinical oncology, surgery and chest medicine. Eligibility criteria included the ability to read and understand English and physical and mental capability to complete questionnaires via a computer touchscreen. Participants were asked to complete, on a touchscreen, the SDI (Wright *et al*, 2005) and the Hospital Anxiety and Depression Scale (HADS) (Zigmond and Snaith, 1983) plus a number of other questionnaires: the Close Persons Questionnaire (Stansfeld and Marmot, 1992), the Mental Health Inventory-5 (Berwick *et al*, 1991) and the European Organization for Research and Treatment of Cancer QLQ-C30 (Aaronson *et al*, 1993). Socio-demographic and clinical information were collected from the medical notes. Procedures for each study were as follows:

Study 2 involves patients at all stages of disease with a range of cancer diagnoses. Patients, recruited consecutively, were asked not only to complete the SDI using a computer touchscreen in clinic and but also indicate whether they thought that they would have benefited from help with each item and overall over the last month. The patients were interviewed at home within a week of completing the questionnaire by a qualified social worker using a structured schedule that covered all areas within the SDI. A total of 189 people took part from 273 approached.

In study 3, 150 newly diagnosed (<3 months from diagnosis) cancer patients treated with curative intent completed the questionnaires and the additional questions concerning the benefit of help in clinic on a touchscreen computer. In all, 36 people declined to take part. Follow-up questionnaires on paper were mailed at 6 months, 12 months and 24 months. The 24-month follow-up is ongoing.

### Nonparticipants

Ethical approval was given in all three studies for data to be collected from the medical notes on those who declined to take part in the studies with no further consent required. These data consisted of age, postcode, sex diagnosis, date of diagnosis and stage of disease.

### Social difficulties

We used the SDI to assess the level of social difficulties experienced by cancer patients. The SDI has demonstrated good reliability and validity and details of the development and evaluation of the SDI are published elsewhere (Wright *et al*, 2002, 2005). The items included on the SDI cover the patient's perception of a wide range of everyday difficulties commonly experienced by cancer patients including difficulties with independence, domestic chores, personal care, care of dependents, support for dependents, welfare benefits, finances, financial services, work, planning the future, communication with those close to you and others, plans to have a family, sexual matters, body image, isolation, mobility, where you live, recreation and holidays. Scoring is on a 4-point scale with responses spanning 0=no difficulty, 1=a little difficulty, 2=quite a bit of difficulty and 3=very much difficulty.

### Psychological distress

The HADS was used to evaluate the level of psychological distress reported by the participants. This is a 14-item questionnaire designed specifically for detecting anxiety and depression in physically ill people and has been shown to be a valid instrument for use in oncology practice using touchscreen technology (Cull *et al*, 2001).

### Deprivation

Carstairs and Morris scores derived from census data from the 1991 census were used to classify small postcode sectors (Carstairs and Morris, 1991; Census Dissemination Unit, 2004).

### Sociodemographic. and clinical variables

Information on age, sex, postcode, diagnosis, date of diagnosis and stage of disease was collected from the medical notes.

### Statistical analysis

The *χ*^2^ test and *t*-tests were used to look at differences between participants and nonparticipants. Two deprivation groups, using the median Carstairs and Morris Index rate as the cutpoint, were created resulting in one more affluent group (−4.51 to −0.86) and the other more deprived group (−0.77 to 12.44) and labelled as ‘affluent group’ and ‘deprived group’.

We used univariate analyses of variance to explore the impact of age (three groups: ⩽40, 41–60, ⩾61), sex, stage of disease (six groups: see Table 1) and deprivation (two groups: see Table 1) on social difficulties (sum of the SDI scores) and on psychological distress (sum of the HADS scores). To examine associations we undertook *χ*^2^ tests and calculated odds ratios using the same groupings described in the univariate analyses. The SDI and HADS scores were split into two groups based on the median sum scores. For the SDI, a low scorers group (sum score 0–8) comprised 54.8% of the sample and high scorers group (sum score 9–50) comprised 45.2% of the sample. The HADS median sum score fell between 12 and 13, coincidentally in line with Razavi's suggested screening cut point of 13 for identification of cancer patients experiencing major depressive or adjustment disorders (Razavi *et al*, 1990). This resulted in the two groups comprising low scorers 0–12 (45.1% of the sample) and high scorers 13–33 (54.9% of the sample). Analyses were performed using SPSS and Excel.

## RESULTS

### The sample

A total of 842 people were approached, of whom 609 consented and 233 refused (participation rate of 72.3%). People who consented to take part were on average younger (*t*=−3.723, df=840, *P*<0.0001) than in the refusing group and men were more likely to participate than women (*χ*^2^=5.134, df=1, *P*=0.023). No differences were found between the groups in terms of disease stage. Of the 842 patients asked to participate, Carstairs and Morris scores could not be provided for 43 postcodes (consenting group *N*=33, refusing group *N*=10). People who refused to take part in the study were more deprived than those who participated (*t*=−2.548, df=797, *P*=0.011).

Details of those who consented are shown in Table 1.

### Social difficulties

There was a statistically significant difference between social difficulties scores for the different groups (F (67, 576)=2.4, *P*<0.0001) with stage of disease (F (5, 576)=7.6, *P*<0.0001), age (F (2, 576)=4.8, *P*<0.009) and to a lesser extent deprivation (F (1, 576)=4.0, *P*<0.048), making a significant contribution. Younger people, those with more advanced disease and those from more deprived areas reported more social difficulties than other groups. One statistically significant interaction was observed between stage of disease, deprivation, age and sex (F (8, 576)=2.406, *P*=0.015). No other interactions reached statistical significance.

**Figure 1:**
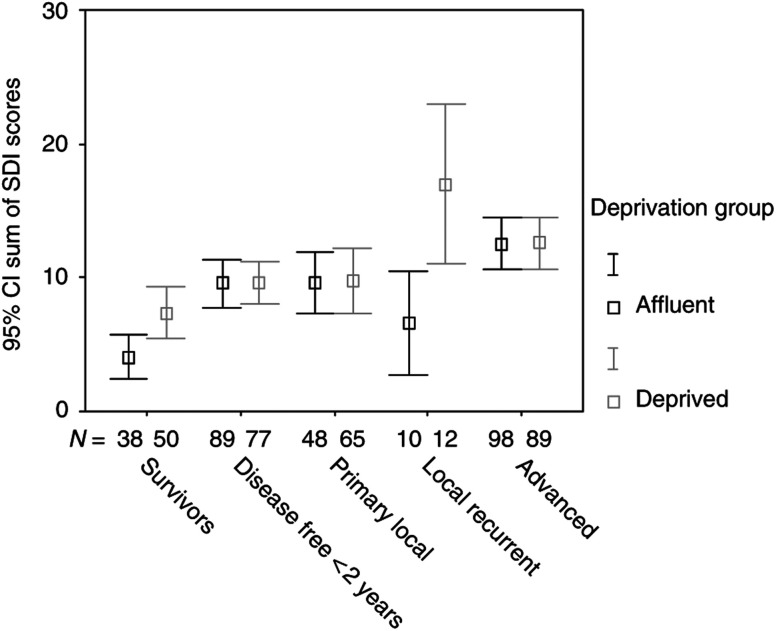
Graph showing mean social difficulty scores with 95% confidence intervals by deprivation and stage of disease. Survivors=disease free diagnosed more than 2 years ago; disease free <2 years=disease free diagnosed less than 2 years ago; advanced=includes people with metastatic disease and those with advanced ovarian and haematological malignancies that cannot be classified using the other categories.

Owing to the interaction found we undertook *χ*^2^ analyses and calculations of odds ratios on subgroups of the sample. Subgroups, defined by age, sex and stage of disease, were used to examine the association between social difficulties and deprivation more closely. The only subgroups that demonstrated significant differences in the level of social difficulties reported in relation to deprivation were for ‘survivors’ (disease free and diagnosed more than 2 years ago) (*χ*^2^=5.835, df=1, *P*=0.016, odds ratio=3.71, CI 1.23–11.19) and for those with locally recurrent disease (*χ*^2^=8.824, df=1, *P*=0.003, odds ratio=20, CI 2.29–175.05), shown in Figure 1. No other significant differences were found.

### Psychological distress

Univariate analysis of variance revealed a significant difference in reported psychological distress between the groups (F (67, 575)=1.723, *P*=0.001). As disease progresses (F (5, 575)=3.267, *P*=0.006), for women (F (1, 575)=4.597, *P*=0.033) and with greater deprivation (F (1, 575)=4.930, *P*=0.027) psychological distress was increased. There were no statistically significant interactions observed.

## DISCUSSION

It is reassuring that we confirmed that psychological distress in cancer patients is associated with deprivation as has been shown by others. This was also found to be the case for social difficulties but to a lesser extent with the stage of the cancer and the age of the patient having a greater impact on the reported social difficulties.

Finding that perceived social difficulties are not more closely associated with social deprivation across a wide range of socially diverse communities is perhaps surprising. It implies that the perception of difficulties detected by the SDI reflects the patients’ tendency to report issues that they believe may be related to the ‘matter in hand’ that is, the diagnosis and treatment of cancer. Underlying social deprivation may not be perceived as a source of social difficulty, simply background against which current issues need to be considered. Cancer and cancer treatments at all stages have a social impact on all patient groups, irrespective of socioeconomic status. Deprivation becomes of greater significance when patients become ‘survivors’, possibly picking up on underlying social issues unrelated to the cancer. It is harder to find an explanation for why people with locally recurrent disease from less affluent areas report more social difficulties than affluent people other than the numbers in this group being much smaller than for the other groups and therefore possibly less representative indicated by the wide confidence intervals shown in Figure 1.

A difference in the level of social problems reported by people from diverse geographical areas was found by Corney and Clare (1985) in a study published in 1985 using a social problems questionnaire developed for use in primary care . The percentage of people reporting a range of social problems was higher for respondents from inner London than outer London. However, a more recent study looking at ward-level deprivation and various aspects of individuals’ social and economic lives suggests that adversity is more closely associated with individual and household characteristics rather than the area of residence (McCulloch, 2001). This is more akin to our work, both in this work and in an earlier study (Wright *et al*, 2002, 2005), where we found age and stage of the disease to be the most significant factors in the reported level of social difficulties.

There are limitations to this work. In our study, we found that more deprived people were less likely to take part, potentially excluding a very vulnerable group of patients. Patient ineligibility may also have played a role with those who could not read or understand English possibly belonging to more deprived groups. Of the 233 people who chose not to take part only once was a ‘dislike of computers’ given as the reason for nonparticipation suggesting that deprivation, confident use of computers and nonparticipation may not be associated. However, this may not reflect the true proportion of people who are uncomfortable with using computers and who choose not to disclose this. In an earlier study, we examined compliance with routine patient-centred assessment in oncology clinics following patients for 6 months. All those who participated used the touchscreen computers. We found that deprivation predicted poorer compliance over time suggesting that issues other than electronic data capture systems influence whether or not people continue with touchscreen assessment (Wright *et al*, 2003).

Patients were recruited from the Cancer Centre only. Information provided by the Northern and Yorkshire Cancer Registry established that cancer patients attending the Centre were more deprived than those who did not attend the Leeds Cancer Centre (*t*=6.043, df=35576, *P*<0.001). Carstairs and Morris scores were not available for 33 people within the consenting group. The reason for this is unclear but may be due to postcodes being allocated to newer housing schemes after the sector scores were derived from the 1991 census.

Health care professionals cannot exclude the possibility of social difficulties being an issue for any patient irrespective of socioeconomic status. It is impossible to identify a high-risk subgroup. To a limited extent, there is some evidence for altering practice concerning psychological distress with indications to support this provided by Macleod *et al* (2004) suggesting that differences in access to information may be a key to levels of distress reported by breast cancer patients. There is clearly more investigation required prior to any recommendations for change being advocated.

## CONCLUSION

Health care professionals cannot predict which cancer patients are at higher risk of social difficulties based on age, gender and social deprivation at different stages of disease. National Institute for Clinical Excellence guidance should be implemented and difficulties assessed using interviews or validated instruments. The information should be given to health professionals for use in consultation with patients and families so that appropriate support can be organized.

## Figures and Tables

**Table 1:** Clinical and socio-demographic information and questionnaire scores of consenting group

**Characteristic**	**Number of participants**
*Age groups*	
⩽40 years	112
41–60 years	262
⩾61 years	235
	
*Sex*	
Males	304
Females	305
	
*Carstairs and Morris index groups* a	
Affluent group (score range −4.51 to −0.86)	283
Deprived group (score range −0.77 to 12.44)	293
	
*Diagnosis*	
Head and neck	53
Lung	47
Genito-urinary	45
Germ cell	60
Haematological	83
Gastro-intestinal	93
Breast	104
Gynacological	70
Sarcoma	19
Melanoma	29
Brain	4
Unknown primary	2
	
*Stage of disease*	
Survivors^b^	94
Disease free (diagnosed within last two years)	176
Primary local	125
Local recurrent	22
Metastatic	143
Other^c^	49
	
*Hospital Anxiety and Depression Scale (HADS)* d	
Low level distress group (sum score <13)	274
High level distress group (sum score ⩾13)	334
	
*Social Difficulties Inventory (SDI)*	
Low level of difficulty group (sum score <9)	334
High level difficulty group (sum score ⩾9)	275

a Carstairs and Morris scores were available for 576 participants (33 missing values).

b Disease free diagnosed more than 2 years ago.

c Includes people with advanced ovarian and haematological malignancies which cannot be classified using the other categories.

d One participant had missing data from the HADS.
